# An Automated Microfluidic Multiplexer for Fast Delivery of *C. elegans* Populations from Multiwells

**DOI:** 10.1371/journal.pone.0074480

**Published:** 2013-09-17

**Authors:** Navid Ghorashian, Sertan Kutal Gökçe, Sam Xun Guo, William Neil Everett, Adela Ben-Yakar

**Affiliations:** 1 Biomedical Engineering, University of Texas, Austin, Texas, United States of America; 2 Electrical and Computer Engineering, University of Texas, Austin, Texas, United States of America; 3 Mechanical Engineering, University of Texas, Austin, Texas, United States of America; Glasgow University, United Kingdom

## Abstract

Automated biosorter platforms, including recently developed microfluidic devices, enable and accelerate high-throughput and/or high-resolution bioassays on small animal models. However, time-consuming delivery of different organism populations to these systems introduces a major bottleneck to executing large-scale screens. Current population delivery strategies rely on suction from conventional well plates through tubing periodically exposed to air, leading to certain disadvantages: 1) bubble introduction to the sample, interfering with analysis in the downstream system, 2) substantial time drain from added bubble-cleaning steps, and 3) the need for complex mechanical systems to manipulate well plate position. To address these concerns, we developed a multiwell-format microfluidic platform that can deliver multiple distinct animal populations from on-chip wells using multiplexed valve control. This *Population Delivery Chip* could operate autonomously as part of a relatively simple setup that did not require any of the major mechanical moving parts typical of plate-handling systems to address a given well. We demonstrated automatic serial delivery of 16 distinct *C. elegans* worm populations to a single outlet without introducing any bubbles to the samples, causing cross-contamination, or damaging the animals. The device achieved delivery of more than 90% of the population preloaded into a given well in 4.7 seconds; an order of magnitude faster than delivery modalities in current use. This platform could potentially handle other similarly sized model organisms, such as zebrafish and drosophila larvae or cellular micro-colonies. The device’s architecture and microchannel dimensions allow simple expansion for processing larger numbers of populations.

## Introduction

*Caenorhabditis elegans* (*C. elegans*) is a powerful model organism in molecular biology due to its simple anatomy, highly conserved and fully sequenced genome, amenability to various molecular and genetic manipulations, and fully characterized cellular anatomy. These attributes facilitated research to the point that the yearly publication rate of *C. elegans* papers doubled during the previous decade [1]. Thanks to their size, geometry, and habituation to liquid environments, these organisms are easily cultivated in well plates using robotic liquid-handling systems. These worms can be subsequently characterized in high-throughput optical sorting systems, such as flow-cell imagers and microfluidic devices. Further advancements and automation in these devices will start revolutionizing drug discovery and high-throughput biology with *C. elegans*, making it an in vivo model with which to investigate complex biological phenomena at speeds and scales only achieved in simpler in vitro studies [2,3]. In this paper, we address a time bottleneck problem in screens across huge numbers of distinct worm populations. Specifically, we present a microfluidic system for ultra-rapid transportation of worm populations treated with different compounds from well plates to the given optical interrogation platform.

The only commercially available sorting system for *C. elegans*, the COPAS Biosort, is a modified flow cytometer that can optically scan and characterize the worms at rate of 100 animals per second [4-6]. This apparatus can obtain single dimensional fluorescence intensity and optical density data from each animal. Before being sent to the optical elements, worms are housed in one large suspension or as separate populations in the reservoirs of a well plate. For screens with many distinct populations, the system also utilizes mechanical suction through tubing to transport the organism populations from well plates to the imaging hardware and requires ~45 sec/population. This timing is necessary to remove the bubbles introduced to the sample during the tubing’s periodic exposure to air. Bubbles can obstruct the field of view for imaging and generate artifacts in high-throughput data collection. This bubble-removal makes the sample delivery time more than forty-fold longer than the imaging and sorting steps.

Several research labs, including our own group, have been developing higher resolution optical imaging and manipulation platforms for *C. elegans* bioassays with microfluidic approaches [7-25]. These devices are generally designed in serial or parallel formats. In the parallel platforms, multiple animals can be housed and monitored simultaneously for analysis of development and life span [9,17,18,21]. Serial loading chips transport and process the animals one-by-one for high resolution imaging, sorting, or optical manipulation [11-15,19,20]. In these microfluidic devices, sample populations are manually loaded with syringes. While this approach is simple and requires no special equipment, it can be very cumbersome and slow (up to ~15 min) [9]. Syringes need milliliter volumes to address the nanoliter-scales of microfluidic devices without introducing bubbles. This requirement could lead to excess consumption of precious reagents and necessitate more effort and resources to prepare extra animals for screens. Researchers have also used a mechanical suction method analogous the COPAS system’s delivery mechanism to send *C. elegans* from well plates to a microfluidic device built for laser axotomies [19]. They frequently observed bubble and debris contamination, necessitating an additional on-chip washing step for each worm surgery.

Biological screens, especially at the larger scales on the microfluidic and commercial platforms discussed would be substantially enhanced by an ultra-fast delivery platform for receiving specific worm populations. Such a platform should easily interface micron-scale fluidic environments to the macro-world, where worm populations are prepared with liquid-handling robotic systems. Furthermore, it should automatically deliver the populations without cross-mixing or adding bubbles. Microfluidics readily meets these criteria. The technology enables liquid-liquid connections between platforms without air exposure, reducing bubble contamination. These aspects are important for the stability of fluid flow profiles and error-free data collection. Additionally, built-in microfluidic valves controlled via multiplexing can streamline automated device functionality [26,27].

Valve multiplexing enables exponentially complex function with fewer pneumatic inputs, leading to increasingly smaller devices. For example, binary multiplexers can regulate “*n*” separate sample channels with 2×log_2_(*n*) pneumatic microfluidic control valves (e.g. 20 valves regulates 1024 samples) [26], and another combinatorial multiplexer scheme is even more efficient and uses “*N*” control valves to regulate N! /(N/2)^2^! individual sample channels [28]. To date, microfluidic devices with multiplexed valve control are predominantly designed for handling liquid compounds in chemical, biochemical, and cell-based studies [26-32]. However, such samples behave much more predictably than freely moving worms or other small organisms. More recently, it was suggested that a microfluidic multiplexer could deliver chemical compounds from standard well plates to multiple *C. elegans* inside the device [11]. Yet, a microfluidic platform for the actual delivery of live microorganism populations (e.g. cell clusters, nematodes, drosophila larvae, and zebrafish larvae) has never been demonstrated. Such a device would need to address the complications of repeatedly transporting populations of motile multicellular organisms in microfluidic channels, preferably without harmful anesthetics.

This paper describes a microfluidic multiplexer device, the *Population Delivery Chip*, which can rapidly deliver 16 different worm populations to a desired location without cross-mixing or introducing any bubbles to the samples, and the system does not affect the worms’ viability. The platform is capable of delivering each population to a prescribed location in 4.7 seconds, a nearly ten-fold increase over current platforms. The *Population Delivery Chip* has conical on-chip reservoirs arranged in a well plate format and spacing, making the device compatible with high-throughput robotic liquid-handling systems. In the following sections, we will present device design considerations and optimization efforts of the microchannel geometries, valve architectures, well plate reservoir interface, and the automated population delivery sequence. With *C. elegans* as the experimental model, the system could significantly reduce the time needed for large-scale drug screens and enhance fluid-based assays for a variety of applications.

## Materials and Methods

### Device Fabrication

**Figure 1:**
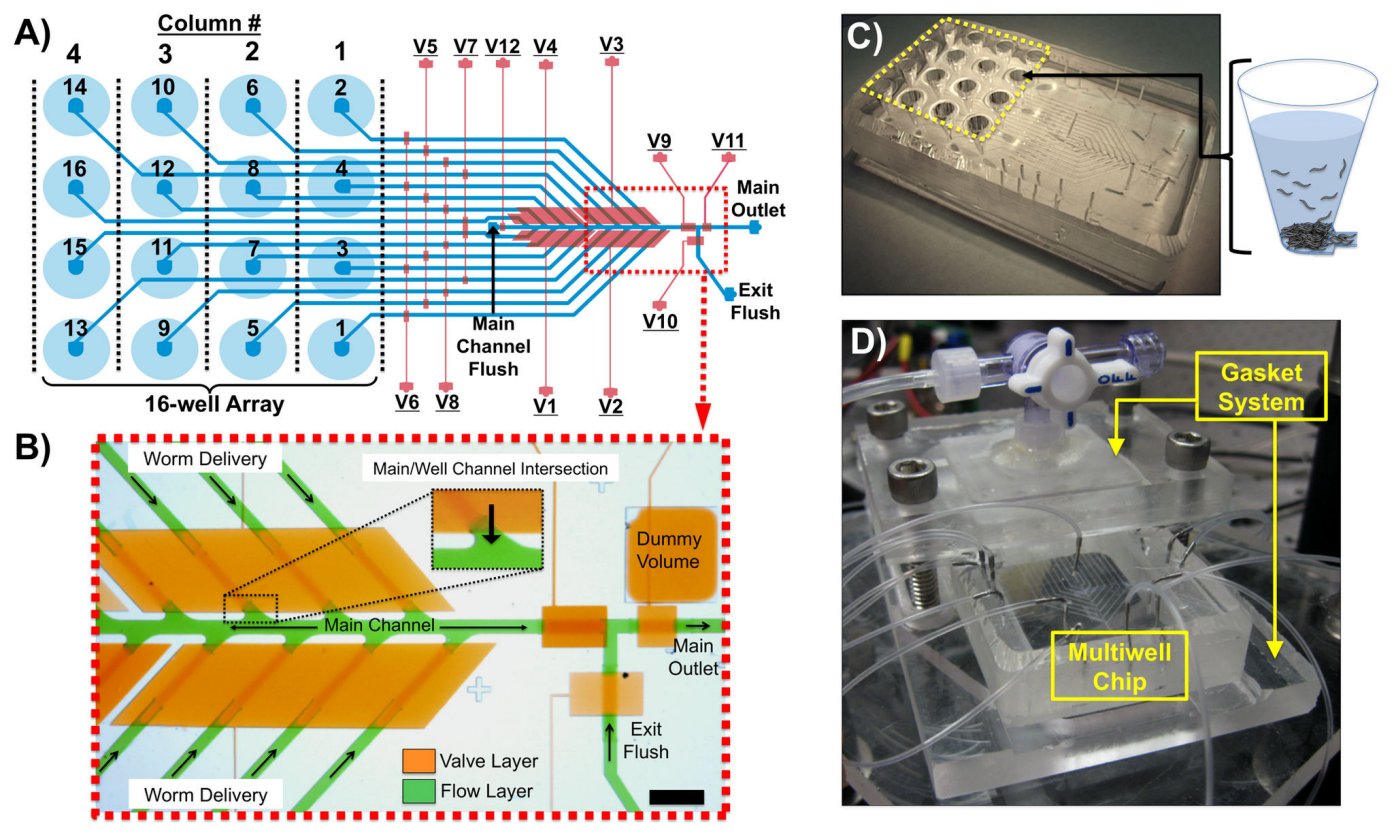
*Population Delivery Chip* design. A) A schematic of the device indicating the flow layer (blue) and control valve layer (red). There are 16 on-chip wells arranged in a 96-well plate format for initial loading of different worm populations. Columns and wells of the array are numbered according to order of delivery. Valves *V1-V8* are multiplexer control valves and *V9-V12* control flow in the main channel. B) An image of the device with its microfluidic channels loaded with food coloring dye, showing the flow layer (green) and control valve layer (orange) (scale bar ~1mm). C) A macro-scale view of the device with the 16-well array indicated by the yellow dashed lines and a schematic of worms loaded into one of the conical wells. D) A macro-scale view of the entire chip/gasket system with pressurized input lines in the experimental setup.

We used modified multilayer soft-lithography techniques for the fabrication of our devices [29]. Two photoresist molds on silicon were used to pattern the two layers of microchannels in the device. The “flow layer” (Figure 1A, blue), which contained the worms, required two photolithography masks to make the positive photoresist (AZ50-XT, Applied Electronic Materials Inc.) features and the negative photoresist (SU-8 2025, Microchem Corp.) features. The average height of the “flow layer” was ~55 µm. A dummy volume was patterned around main exit channel to prevent over-development of portions that were patterned in positive photoresist (Figure 1B). We also patterned the control layer mold in the negative resist (~36 µm in height).

We then patterned features into the elastomer (polydimethylsiloxane, PDMS, Dow Corning) with these molds. PDMS was mixed at a 10:1 (resin: crosslinker) ratio and poured onto the flow layer mold, which had conically-shaped p-1000 pipette tips pre-positioned on the well entrance ports by a PMMA holder surrounding the wafer (Figure S1). The conical wells could hold up to 100 µL of fluid depending on the height of the device. A ~55 µm thick layer of PDMS was then spin-coated onto the control layer mold to create on-chip valves. We placed this layer with the mold in a 65°C oven until the spin-coated PDMS was partially cured. The PDMS flow layer piece was then bonded on top of the control layer via oxygen plasma treatment and sat in the 65°C oven overnight for bond strengthening. Though not absolutely necessary, oxygen plasma treatment enhanced bonding between the flow layer piece and the partially cured surface PDMS on the control layer mold. This treatment allowed for longer curing of the control layer’s PDMS prior to bonding to reduce collapses of the flow layer channel ceilings onto the control layer mold’s PDMS coating. After this process, we bonded the entire chip to a sheet of 3/16 inch thick borosilicate glass via oxygen plasma treatment. The device was then left in 65°C for at least 4 hours to enhance the glass to PDMS bonding.

### On-Chip Valve Control

The pneumatic control setup consisted of 16 three-way solenoid valves (The Lee Company) controlled through a voltage amplifier (ValveLink 8.2, AutoMate Scientific, Inc.) and a NI-DAQ 6501 controller board (National Instruments Corp.), which was connected to a computer via USB port. We fed DI water (valves) and M9 solution (flush channels) from air-pressurized reservoirs into the on-chip fluid inlets through the solenoid valves. When they were in the “off” or open position, the inputs leading to on-chip valves were exposed to a vacuum of -8.7 psi (~-60 kPa) gauge pressure via the solenoids. The vacuum pressure increased the valves’ opening speed and led to faster on-chip responses to the automated control program.

We developed automation software in LabVIEW to control actuation of the solenoid valves. A separate image acquisition program provided by the camera manufacturer (Manta G201 IRC, Allied Vision Technologies) captured video and images during experiments.

### Device Fluid Priming

After securing all of the fluidic connections on the device, we loaded 100 µL of M9 solution into each on-chip well. We then sandwiched the device in the gasket and sealed it to the device’s well plate reservoirs by uniformly tightening the four screw clamps (Figure 1D). We primed the device for experiments by first introducing fluid from the flush channels until there were no longer any bubbles coming into chip. We then opened all of the on-chip valves excluding those that blocked off the flush channels (these channels eventually led to fluid reservoirs) and pressurized the wells at 7.5-10 psi (~50-70 kPa) through the gasket. Once fluid had completely filled the microchannels, valve *V11* (Figure 1A) was closed to block flow out of the chip, while pressure was still applied to the gasket. This pressurization forced the remaining air bubbles to diffuse out of the fluid and into the surrounding PDMS. Generally, within 10 minutes all bubbles were removed from the device channels. All on-chip valves were then closed prior to sample loading.

We then released the chip from the gasket for worm population or fluid loading. First excess fluid was removed from each of the wells until ~10-20 µL of M9 solution remained. For fluidic flow rate experiments the wells were filled with M9 via syringe or pipette. For worm delivery experiments, worms were prepared in M9 suspension and loaded into the device at a density of ~100-200 worms per on-chip well. We then sealed the gasket to the device and for less than 5 minutes for the worms to settle to the bottom of the wells.

### Measurement of Fluid Flow Rates

We primed the chip with M9 solution and then executed an automated valve sequence to send fluid from a specific on-chip well during the application of different gasket pressures. Fluid exiting the chip was collected in a reservoir and weighed on a high-resolution scale (Adventurer Pro AV213, Ohaus Inc.) immediately after sample collection to negate effects of evaporation.

### Worm Culture Techniques

#### Preparation of NGMSR plates seeded with bacteria

Saturated cultures of HB101 *E. Coli* were grown by inoculating 250 mL of LB Broth, Miller (Fisher Scientific) with a single colony and incubating the culture for 24 h at 37°C. We seeded 10 cm NGMSR (Streptomyosin-Resistant Nematode Growth Medium) plates with bacteria by adding 1-2 mL of saturated HB101 to each plate and leaving the plates with their lids closed at room temperature for 2–3 days for drying. The bacteria served as the worms’ primary food source. NGMSR pads were prepared with Nistatin (anti-fungal, Fisher Scientific, 0.01 mg/mL) and Streptomyosin sulfate (anti-bacterial, Sigma Aldrich, 0.2 mg/mL).

We used the following strains in our experiments: SK4005: *zdIs5* [(*Pmec-4::gfp*) + *lin*-15(+)] I, CZ1200: *juIs76* [(*Punc-25::gfp*) + *lin*-15(+)] II; *lin-15b*(*n765*) X, TU3311: *uIs60* [*Punc-119::yfp + Punc-119::sid-1*], and TU3595: *uIs72* [pCFJ90 (*Pmyo-2::mCherry*) + *Punc-119::sid-1* + *Pmec-18::mec-18::gfp*]; *sid-1*(*pk3321*); him-*5*(*e1490*) V; *lin-15b*(*n744*) X.

#### Preparation of synchronous worms

We transferred a large population of gravid adult worms grown on seeded NGMSR plates to a small volume (0.3 mL) of a 1:2 mixture of 5 M sodium hydroxide to sodium hypochlorite (bleach). After 2-3 minutes, the adult bodies are mostly dissolved, leaving unhatched embryos intact. We spun down this suspension with a centrifuge, removed the supernatant, and then added 1 mL of distilled water to wash out the bleaching solution. We repeated the washing step two more times, and pipetted the embryos onto an unseeded NGMSR plate. After 12 hours, most of the embryos reached the L1 life stage. These worms were then transferred to seeded NGMSR plates, where they reached the L4 life stage after an additional 24-28 hours.

#### Preparation of worms for device loading

L4-stage worms were cleaned of debris that might occlude channels in the *Population Delivery Chip* or downstream devices that would receive the animals. Worms were washed off NGMSR growth plates with filtered M9 solution and pipetted into a 15 mL centrifuge tube that was placed in an ice-water bath for 5 minutes. The lower temperature prevented worms from swimming vigorously enough to stay afloat and the vast majority of them sank to the bottom of the tube. We removed the supernatant above the worm pellet and then we refilled it with filtered M9. The process was repeated two more times to minimize bacteria and small debris in the animal suspension.

We then resuspended the worms, loaded 100 µL of the worm stock into a well of a 96-well plate, and counted the population to determine sample density. If adjustment to the suspension density was required, we waited again for the animal stock to sink to the bottom of the centrifuge tube and adjusted the supernatant volume accordingly.

### Worm Viability

For viability tests, animals were scored every 24 hours [33]. The populations were incubated at 16.5°C throughout their lifetimes. The animals were considered dead if they did not move after touch with platinum wire, or crawled off the agar pad. Whenever necessary, we transferred worms to new seeded plates to separate them from their progeny and contaminants. The log-rank test was used to determine statistical differences between test population and control population lifespans. P-values < 0.05 were considered statistically significant.

## Results and Discussion

### Design Considerations

The design process for a microfluidic device for *C. elegans* population delivery required addressing four major design considerations: 1) interfacing with well plate format reservoirs, 2) delivering multiple worm populations without cross-contamination, 3) achieving fast and repeatable worm population size delivery, and 4) hands-free automation within these constraints.

The design considerations culminated in a computer-controlled multiplexed microfluidic device with built-in conical wells for high-speed population loading. The device features an optimized microchannel/microvalve architecture, which facilitates the rapid (4.7 sec/well) and automated delivery of *C. elegans* populations from 16 wells without cross-contamination between populations.

Several design iterations led us to the two-layer microfluidic device presented in Figure 1. The general design includes the following main features: 1) integrated on-chip reservoirs having dimensions consistent with traditional well plates for simple loading of worms into the chip using robotic liquid-handling systems or manual multi-channel pipettors, 2) a multiplexed pneumatic microfluidic valve system to control flow in microchannels emerging from individual wells, 3) a staggered configuration of microchannels merging into the main delivery channel, and 4) two flush units with multiple control valves to accelerate delivery of each worm population to a location of interest and guarantee no animals or chemical reagents remain in areas of the device where mixing can occur.

The integration of wells into the chip substantially simplified the world-to-chip interface. The arrangement of valves near the main channel/well channel intersections, and two device-cleaning flush inlets acted together to prevent population mixing during automated delivery. An acrylic gasket sandwiched the chip to deliver pressurized air to on-chip well reservoirs. Microfluidic valves arranged according to the multiplexer control scheme regulated flow through the device’s microchannels with a minimal number of valve inputs [27]. The automated delivery software allowed for flexible arrangement of valve actuation timings, which were optimized to prevent mixing between populations and achieve fast and nearly complete population delivery during device operation.

### On-Chip Conical Wells for Simple Sample Loading

To address the first design consideration, we integrated an array of well plate format reservoirs within the microfluidic device. A simple molding method enabled us to fabricate identical conical wells into the PDMS and directly connect them to the microchannels in the device (Figure S1). The conical shape of the reservoirs enabled concentrating the animal population at the interface of the well-bottoms and microchannel entrances.

In our earlier approach we made an attempt to interface standard well plates with the microfluidic chip through an acrylic/fluoropolymer gasket (Figure S2A). However, that approach posed a few challenges. The wells interfaced with the chip through metal tubing sticking out of gasket’s bottom side. Aligning all 16 metal tubes with 16 on-chip fluid ports was technically challenging and resulted in sample leakage between the metal tubing and the PDMS chip. Additionally, the population of animals could potentially accumulate in the gasket wells or in the metal tubing coupler and cause contamination.

By integrating the wells directly into the multiplexer microfluidic chip, we could eliminate fluid leakage, simplify initial sample loading, and make the device more compatible with robotic liquid-handling systems. Most worms loaded in fluid suspension would sink and concentrate at the bottom of these conical wells in a couple of minutes; staging them at the well channel entrances before delivery. Having the entire worm population placed at the channel entrance shortened the distance animals traveled on-chip and decreased the timing necessary to deliver a similar number of worms. Wells built into the chip streamlined device operation.

### Design Solutions to Prevent Population Cross-Contamination

Device architecture in relation to microchannel geometries and microfluidic valve location, along with the automated delivery sequence arrangement played major roles in the optimization of the *Population Delivery Chip*’s ability to prevent population mixing during delivery. Occasional cross-contamination between populations within different well reservoirs occurred in our initial designs (Figure S2A). Worms’ unpredictable swimming behavior caused them to periodically deflect at the well/microchannel intersection with the main channel, occasionally directing the worms into other well channels, instead of allowing them to flow out of the main exit (Figure S3). These events could occur despite the fact that the device was directing flow from a single well to the outlet, and not towards the other well channels. We believe that the sharp corners at the diagonal intersections of the well channels and the main channel, and the fact that these intersections were arranged directly opposite of each other (unstaggered) were the two major contributors to cross-contamination. Considering these observations, we pursued a few design solutions that successfully prevented population cross-contamination. The following mechanisms for preventing population mixing also should hold true for potential cross-contamination of chemical reagents incubated with the populations since the on-chip pneumatic valves fully seal when closed.

#### Channel Architecture Solutions to Improve Segregation

We made four key changes to the channel design to prevent worm population cross-contamination between wells. First, we moved the large control valves from an upstream position close to the intersection of the well channels with the main channel (Figure S2B). This design element prevented worms that were moving through main channel from unintentionally being transported into the well channels instead of to the exit. Second, we integrated a flush channel upstream of all of the main channel/well channel intersections to wash out remaining members of a sample population between delivering each population from different wells (Figure 1A). Third, we rearranged the intersection of well channels with the main channel in a staggered pattern to ensure that the entrances of two well channels do not sit directly across from one another along the main channel. Finally, we rounded the sharp channel corners at the intersection of the well channel and the main channel. By eliminating sharp-angled intersections, unpredictable worm movements around these corners were avoided. Automated operation of the final device showed no instances of worms accidently flowing into another well channel (Movies S1 & S2).

#### Automation Sequence Solutions to Improve Segregation

Flow in each well-channel from a given well to the main channel was regulated by an upstream (*V5-V8*) and a downstream valve (*V1-V4*). The sequence for delivery from different wells was chosen based on this valve configuration to prevent cross-mixing between the populations during delivery. Added channel cleaning steps acting together with this delivery sequence helped prevent population mixing as well.

First, we chose to deliver populations in groups of wells that share the same upstream valve (*V5-V8*). By positioning downstream valves close to the main channel, we successfully minimized dead volumes where possible mixing could occur. However, manipulation of freely moving, living organisms required additional attention. Worms could freely swim in the well-channels even if the flow was completely stopped by the upstream and downstream control valves. After transporting most of a given well’s population to the desired location, a few worms could remain in the well channel. We therefore implemented a “flushback” or cleaning step to push any worms remaining in the well-channel back to their destination well, beyond the upstream valves. This step prevented them from escaping into the main channel during delivery from other wells with which they shared the same downstream valve (*V1-V4*). This washing step alone, however, was not sufficient if the order of wells was randomly chosen. Worms could potentially swim past opened upstream valves even if the downstream valves regulating their well channels were closed. For example, delivery from *Well 7*, just after delivery from *Well 1* could cause the following complication: During population delivery from *Well 1*, *V6* needs to be opened, also allowing *Well* 3’ s population to swim closer to *Well* 3’ s downstream valve (*V1*). If we choose to deliver from *Well 7* next, its population could be cross-contaminated with worms from *Well 3* that slipped downstream of *V6* during delivery from *Well 1* (Figure S4). Such events could only be avoided by applying an additional flushback step on *Well 3* before delivery from *Well 7*. However, we eliminated these types of complications by arranging the sequence such that wells regulated by the same upstream valve would deliver their worms in sequence and then by cleaning their well-channels in a single step as one group before proceeding to the next group. For example, fluid flow from *Wells 1-4*, the first column of wells, is regulated by valve *V6*; therefore the optimized sequence delivered populations from *Wells 1-4* consecutively (Movie S2). Unloading wells in this fashion also minimized the number of washing sequences required. Specifically, after delivering all four of these populations, the automated program performed a single cleaning step to wash back any excess worms in the well-channels to their respective sample reservoirs. Cleaning steps did not need to be repeated on these wells since *V6* never reopens.

Secondly, the chosen order of well columns unloaded assured that wells whose channels intersect furthest downstream in the main channel had their populations delivered earliest in the sequence. Specifically, the sequence began with *Wells 1-4* in column 1 and proceeded until populations in *Wells 13-16* in column 4 were delivered last. Based on the schematic in Figure 1A and Movie S2, it is apparent that a specific population of worms traveling in the main channel towards the *Main Outlet* could not contaminate any undelivered population as it passed by the downstream well channel intersections of other wells. Either the downstream well channels were already emptied if they shared the large open valve near the main channel with the chosen well channel, or they were not yet emptied but closed off from the main channel by one of the other large valves. Independently, with the new configuration of staggered channels we never observed worms unintentionally entering a well channel.

### Automated and Fast Worm Population Delivery

To achieve the fastest worm population delivery rates, prevent population mixing during delivery, and preserve worm viability, we optimized the delivery pressures and timings. To estimate minimum timings for full population delivery, we first measured flow rates across the range of allowable pressures applied to the gasket, the on-chip valves, and flush inputs. We then tested and adjusted these timing/pressure combinations until we reached nearly full population delivery from each well during the automation sequence. Finally, we verified that the optimized design and delivery sequence could indeed provide fast delivery without population mixing or damage to worm viability.

#### Flow Rates

We measured flow rates across the individual wells to the *Main Outlet* for different gasket pressures ranging from 2.5-20 psi (~17-138 kPa), at 2.5 psi increments (~17 kPa) and compared them to theoretical calculations based on the flow resistance imposed by the individual well channel geometries [34]. Data was collected from one well in each vertical column of the well plate reservoir array.

Measured flow rates generally varied linearly with applied pressure (linear regression, *R* ^*2*^ = ~0.99 for all measured data) and overlapped with the theoretically expected values within 10% (Figure S5). Since fluidic resistance is directly proportional to channel length, the longer channels had lower flow rates given the same pressure at the gasket. This result implied that one would have to linearly adjust the pressures or timings applied to the wells via the gasket system to deliver the same number of worms across wells with different channel lengths. Such differences could be easily accounted for in the automation software, by adjusting the timings of the automation steps for each well.

Sources of slight deviations from theoretical flow rates were most probably a result of the elastic properties of PDMS. At the higher pressures, it is possible for the PDMS channel cross-sections to expand and effectively decrease the channel’s fluidic resistance [35]. This expansion might have been the cause for the slightly higher-than-predicted flow rates observed above 12.5 psi (~86 kPa) in *Wells 2* and *8*. Other sources of small drifts from theoretical values could include physical inconsistencies at the external interfaces to the microchannels caused by the variability of manual punching or the slight accumulation of debris in the device.

#### The Automation Sequence

**Figure 2:**
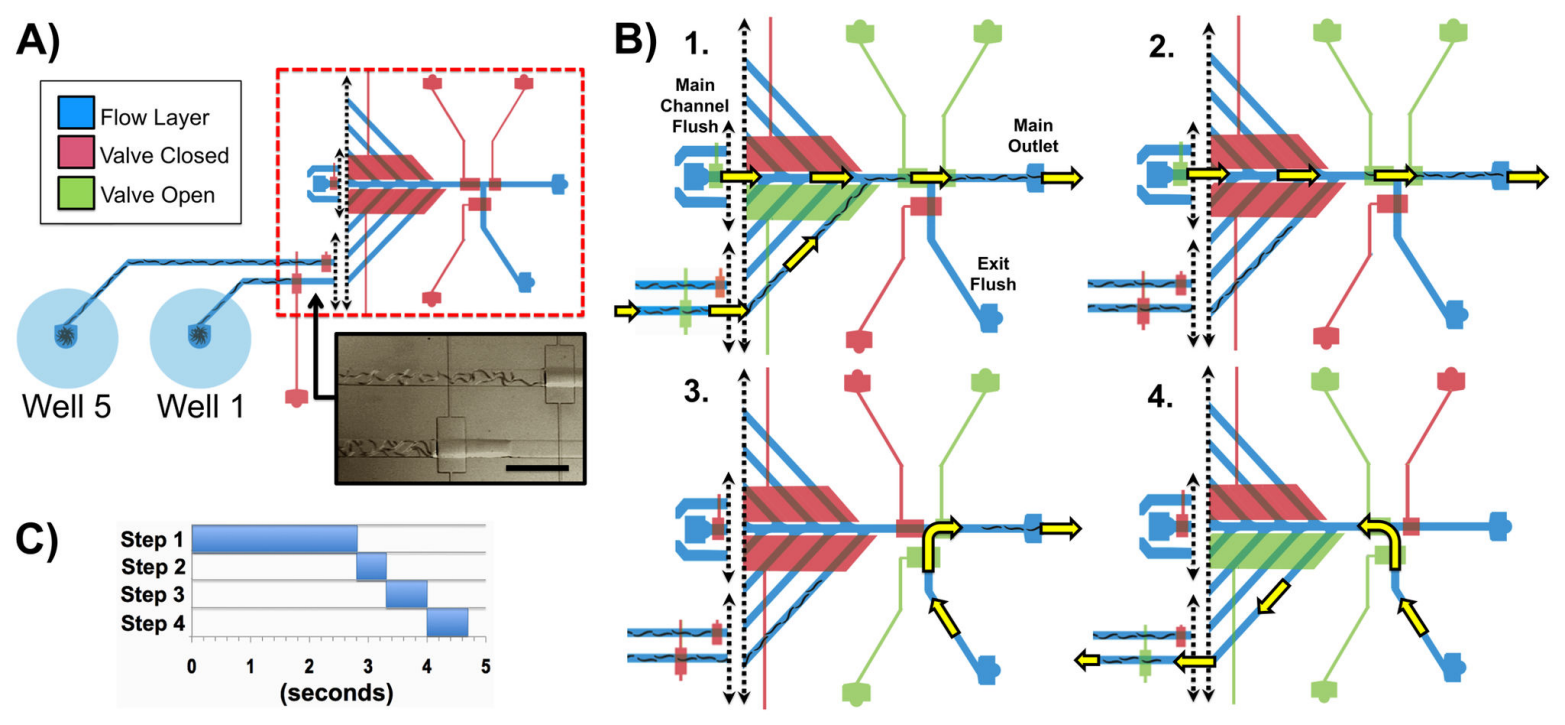
Automated worm population delivery sequence. A) Schematic of the device showing areas active during the sequence example as the worms are pre-staged at the first set of control valves. An image of pre-staged *C*. *elegans* worms is below the schematic (scale bar is 1 mm). B) Illustration of all steps for one full sequence cycle. Step 1: Appropriate valves open as the gasket is pressurized to send *Well* 1’s population to the main channel, where *Main* *Channel* *Flush* then accelerates the worms’ transport to the main exit. Step 2: Excess worms are cleared from the main channel towards the *Main* *Outlet* via flow from *Main* *Channel* *Flush*. Step 3: Flow from *Exit* *Flush* delivers the worms from the *Main* *Outlet* to an off-chip location. Step 4: “Flushback”; *Exit* *Flush* flow is redirected backwards to clear any remaining worms in the well channel back to *Well 1*. This step is executed on *Wells 1-4* only after finishing Steps 1-3 on each of them. C) Timings for each step.

We optimized the delivery sequence to shorten the delivery time of the full population from each well, while still preventing population mixing during delivery. Figure 2 illustrates the steps of the automation sequence and Table S1 presents the optimized timings and the on-chip actuation scheme used in each step.

Within a few minutes after loading the worms in the wells and sealing the gasket, worms begin swimming to the first valve nearest to the wells, which are completely sealed to prevent worm passage (Figure 2A). The delivery of worms then proceeded in four steps with an optimized timing for each step (Figure 2C, Table S1). Figure 2B illustrates the steps of the automation sequence for *Well 1*, as an example. First, the system delivered a single population of worms out of the given well towards the *Main Outlet*. It then flushed excess worms from the main channel and completed their delivery to the location of interest by using flow from *Exit Flush*. Finally, it sent undelivered worms back to their origin well. Since *Exit Flush* had an essentially limitless fluid reservoir and faster flow rate per unit pressure than any of the well channels, it could quickly complete worm population delivery, which minimized the time spent driving flow through the origin well. This strategy eliminated the risk of exhausting the well’s fluid supply and introducing bubbles to the device.

Further sequence optimization could include the execution of Steps 3 and 4 simultaneously. Since *V9* is closed and *Main Channel Flush* is idle during Step 3, flow from *Main Channel Flush* can be directed back to the origin well to complete the well channel-cleaning step, while *Exit Flush* completes delivery of the original population. This approach will effectively eliminate Step 4’s timing allocation for each well, reducing the overall delivery time for each well to 4 seconds.

#### Timing Optimization

We optimized the timings of the automation sequence by placing worm suspensions (~100-200 worms/well, strain: SK4005) that had reached the L4 life stage into the wells and running the automation sequence to induce flow through the wells at various pressures and timings. We collected the worms delivered during the automated sequence in a well plate. Worms that did not get delivered during the sequence were collected immediately afterwards. The two groups were counted for each well in order to determine the total number of worms initially loaded in a given well relative to the total delivered during the sequence.

Considering the measured fluid flow rates (Figure S5) and well channel distances, we postulated adequate timings for the full population delivery from each well for a given pressure. Based on these calculations and preliminary experiments, we found a timing of 2.8 seconds for Step 1 with the maximum pressure (20 psi, ~138 kPa) applied through the gasket, would be adequate to empty the slowest wells in the device. Thus, we applied this timing to all of the wells to ensure maximal delivery success across the chip at the highest pressure. These timings and the measured flow rates also indicate that the fluid volume delivering the population would be three orders of magnitude larger than the volume of the worms’ bodies (for ~150 animals). This volume ratio suggests that the worms would not spatially overlap with each other inside an imaging platform.

#### Fast Worm Population Delivery

To characterize the robustness of the population size delivery across different pressures at the chosen timing, we measured the number of delivered worms for various gauge pressures to the gasket and the *Main Channel Flush* fluid reservoir (5-20 psi, ~35-138 kPa) but maintained a 20 psi gauge pressure for the *Exit Flush* reservoir throughout the experiments. The conical cross-section of the wells enabled a majority of the worms loaded into a given well to settle at the base of the sample reservoir, close to the well channel entrance or the first valve (Figures 1C and 2A). With the conical shape of wells and optimized timings in the automated delivery sequence, we achieved quick and nearly complete delivery of the worm populations from the on-chip wells to an off-chip location.

**Figure 3:**
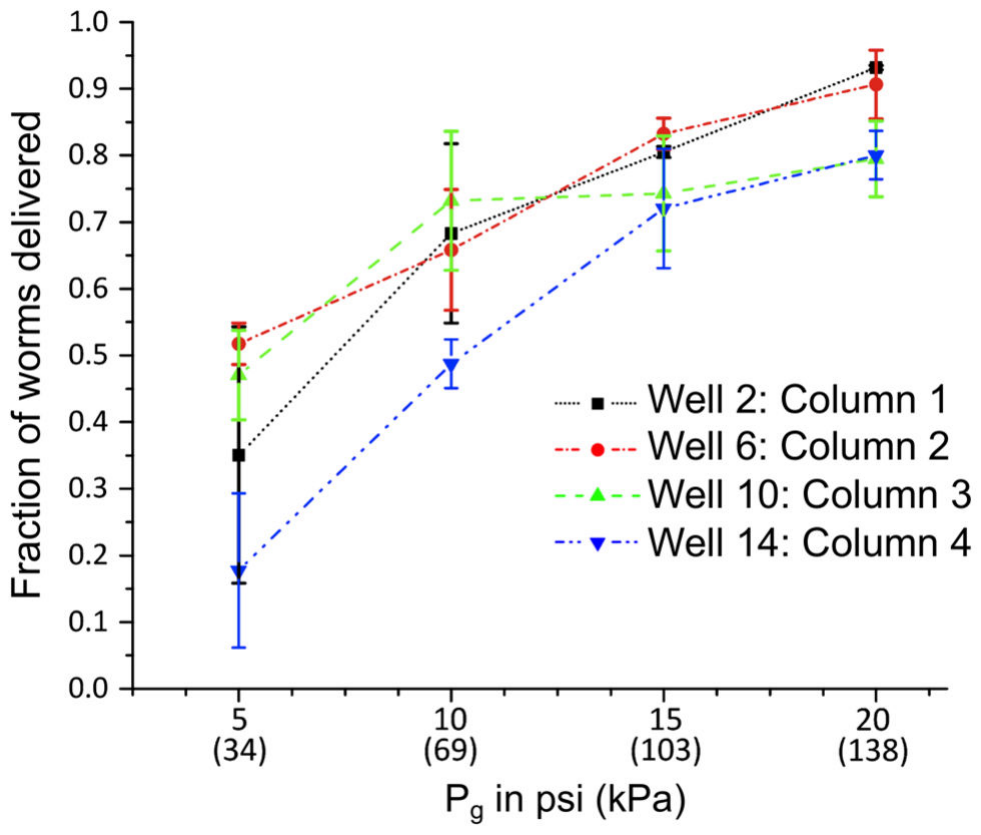
Worm population delivery as a function of applied pressure. The fraction of worm populations loaded in 4 representative on-chip wells from 4 different columns of the *Population* *Delivery* *Chip* that are delivered to the outlet of the device as a function of pressure applied at the gasket and the *Main* *Channel* *Flush*.

Figure 3 shows what fraction of the initially loaded worm populations in 4 wells were delivered for various gauge pressures applied to the gasket and the *Main Channel Flush*. At the maximum pressure applied to the gasket and *Main Channel Flush* (20 psi, ~138 kPa), the *Population Delivery Chip* only required 4.7 seconds per well to deliver an average of 80-93% of each worm population initially loaded into the on-chip wells. Delivery of more than 100 worms from each population is more than adequate for most *C. elegans* bioassays. The achieved delivery speed took place in a fully automated process and was nearly an order of magnitude faster than any other platform capable of delivering worm populations from wells without bubbles (45 sec vs. 4.7 sec) [4,5]. Saving 40 seconds of transport time per population would potentially eliminate 40 million seconds of actual experimental time when applied to a million-compound screen. This time is equivalent to 1,388 days (8 hrs/day), 277 weeks (5 days/week), or 5.55 years (50 weeks/year) of time savings for a single large screen.

Since a majority of the worms settled at the bottom of the wells before delivery, most of a population moved from its well to the main channel after only a fraction of the well reservoir volume was exhausted. Consequently, the fluid level in a given well was maintained near its initial value and bubbles could not enter the device via exhaustion of the reservoirs. If desired, we could create actuation timings that nearly but not completely exhaust the wells’ fluid supplies without bubble introduction since the flow rates were well characterized. We could further improve the overall delivery speeds and efficiency by reducing overall channel lengths and increasing channel cross-sectional dimensions to reduce flow resistances, and applying higher pressures to the gasket and flush channels.

#### Experimental Validation of Population Segregation

We next performed experiments to confirm that the automation and device architecture maintained worm population segregation. Instead of using 16 different populations, we used only 4 strains and ran two different tests. In the first test we loaded each row of the well plate reservoir array with a different *C. elegans* strain, and then in the second test we loaded each column with a different strain. Each of the 4 strains had a different set of neurons labeled with endogenous fluorescent markers, making visual differentiation between the strains via microscopy very simple. We placed one of the 4 strains into a set of 4 wells and ran the automated delivery sequence at the maximum operational gauge pressure (20 psi, ~138 kPa). We then collected the populations in 96-well plates and observed each worm collected on a fluorescent stereoscope to characterize their neurons’ morphologies and confirm strain type. We also counted the number of worms belonging to each strain within the collected population samples.

**Figure 4:**
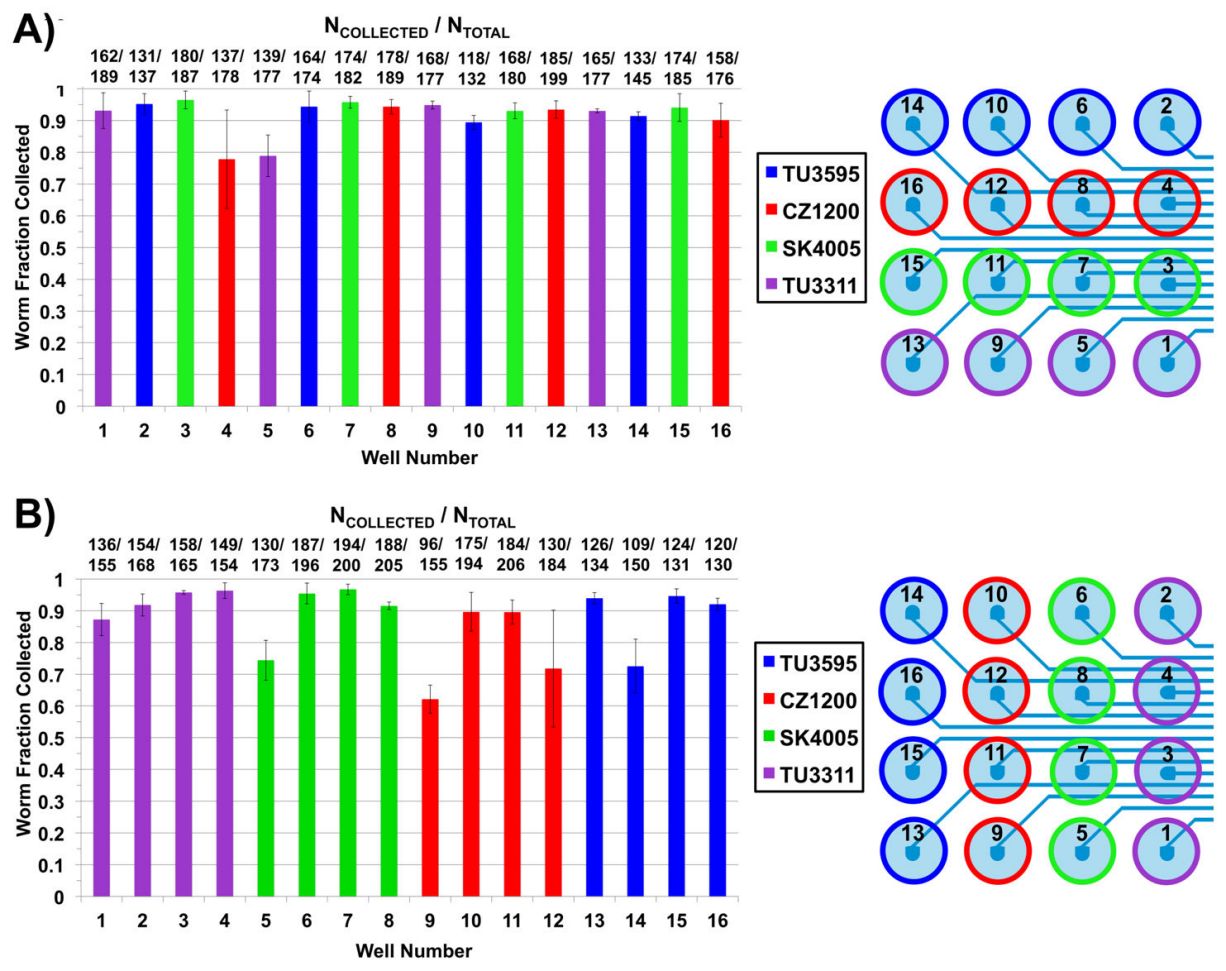
Population mixing eliminated during automated delivery at 20 psi (~138 kPa). The graphs show the fraction of animals collected after delivery from a given well that are of the same strain initially loaded into the well. The actual average number of collected worms over the average number of those initially loaded is indicated above each bar. A) Four distinct strains loaded in each **row**. B) Four distinct strains loaded in each **column**. A corresponding color-coded schematic on the right of both graphs indicates into which wells the strains were loaded at the beginning of both experiments. Each color represents a single type of strain.

The data shown in Figure 4 suggest that a synergy of the automated delivery sequence and microchannel architecture prevented population cross-contamination between wells during operation of the *Population Delivery Chip*. With each row of the well-reservoir array having a different *C. elegans* strain (Figure 4A), the device only delivered worms of the expected strain for each well. This result implied that there was no mixing between the rows of the well array. However, mixing between columns was not yet ruled out, since all four wells in each row had the same strain. To confirm the elimination of mixing between columns, the experiment was repeated with a distinct strain loaded in the 4 wells of each column instead of each row (Figure 4B). These experiments definitively demonstrated the device’s ability to deliver 16 different populations to a desired location without cross-contamination.

#### Animal Viability after Delivery through the Device

To test the effect of chip delivery on worm viability, we loaded SK4005 worms into 4 representative wells on the device and delivered them to the main exit port with the automated delivery sequence. We randomly collected 20 worms from 4 wells for each condition tested and put each population onto separate NGMSR growth pads. For control tests, we also collected 20 worms from the original worm suspension, which never experienced on-chip delivery.

**Figure 5:**
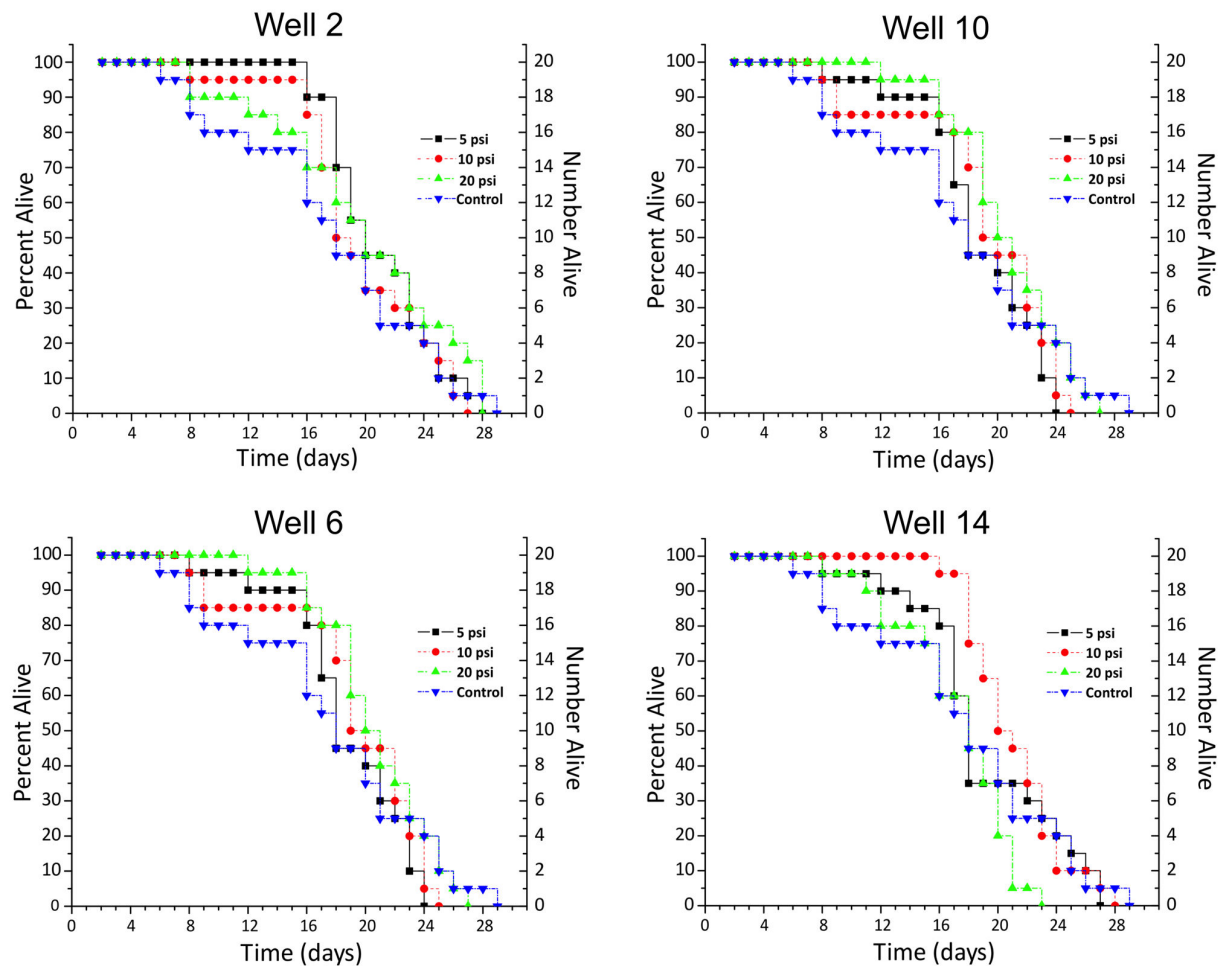
Worm viability test. The daily change in live worm population numbers for 3 different applied pressures to the gasket relative to control in four representative wells; each well resides in a different column of the device.

Figure 5 presents the day-by-day survival data of 20 worms after delivery through the chip with various pressures (5 psi, 10 psi, and 20 psi) applied to the gasket. When compared to the control group, differences in animal viability for 11 out of the 12 groups delivered through the device were not statistically significant. In all cases, every single worm survived at least 6 days and every population had at least one worm that lived between 23-29 days.

Table 1 further summarizes the total average lifespans of worms in the test groups and the log-rank test statistics comparing them to the control group. Looking at the average lifespan of worms in our control group (17.8 ± 6.6 days) there were almost no major decreases in average lifespans in worm populations delivered via the *Population Delivery Chip*. Among 12 viability tests, only the 20 psi (~138 kPa) group of *Well 14* showed a statistically significant difference. However, as can be seen from Figure 5, the drop off in viability relative to control begins after day 14, which could be attributed to factors such as worm handling during plate transfers. These results implied that the chip would not significantly damage the animals, and the device platform could be a useful component in automating *C. elegans* bioassays across its entire operational pressure range.

**Table 1:** Average animal lifespan (days) after delivery through the whole chip under different pressure conditions.

**Pressure**	**5 psi (~35 kPa**)	**10 psi (~69 kPa**)	**20 psi (~138 kPa**)
**Well Number**	**Life span (p-value**)	**Life span (p-value**)	**Life span (p-value**)
**Well 2**	19.1 ± 3.7 (0.975)	17.8 ± 4.6 (0.447)	17.9 ± 6.3 (0.880)
**Well 6**	16.8 ± 4.1 (0.096)	17.2 ± 5.1 (0.366)	18.7 ± 3.8 (0.679)
**Well 10**	17.4 ± 4.3 (0.399)	17.3 ± 4.7 (0.352)	17.2 ± 6.1 (0.642)
**Well 14**	17.1 ± 5.1 (0.399)	19.2 ± 3.2 (0.956)	15.2 ± 3.9 (*0.036)

The initial size for each worm population was *n*=20. The average lifespan of worms in the control group was 17.8 ± 6.6 days (± indicates standard deviation). The p-value for each condition based on the log-rank test statistic relative to control is given as well. A p-value <0.05 was considered significant.

Indicates statistical difference

## Conclusions

We designed, characterized, and optimized the *Population Delivery Chip* with integrated conical wells for delivery of *C. elegans* worms to a given automated fluidic apparatus. The device could deliver up to 16 different worm populations in sequence to a desired location from its 4×4 array of well plate format reservoirs without mixing any populations, harming the animals’ viability, or requiring anesthetic treatments on the animals. The automated software control produced repeatable flow rates and sizes of delivered worm populations from the device. Furthermore, the platform displayed throughput not seen in other more complex worm population delivery platforms. These advances will accelerate biosorter screens and provide a dependable macro-scale interface for delivering small organisms to automated microdevices for high resolution imaging and manipulation.

In future versions of the chip, initial loading of animal populations could be processed by robotic liquid-handling systems by pressurizing the on-chip pneumatic microfluidic valves to block flow in the well channels. These robotic platforms can handle culturing, preparation, and incubation of worm populations before loading into the delivery chip. Further optimization of channel dimensions would ensure that all channels leading to the wells would have the same fluidic resistances, thus ensuring the same flow rates and worm delivery rates across each well for the same pressures applied to the gasket and fluid inputs. Higher pressures coupled with larger channel cross-sections will allow much faster delivery times, potentially shorter than 2.5 sec/population. Expanding the number of precisely regulated well reservoirs by an order of magnitude can be achieved by adding a few additional control valves to the set of multiplexed valves and rearranging the well channels’ placement and geometry to accommodate more samples. Finally, overall workflow could be sped up by pre-priming many of the microfluidic chips with fluid in parallel inside of a vacuum chamber to remove air bubbles and sealing the chips for future use.

## Supporting Information

Figure S1 **Fabrication of well plate reservoirs for the *Population Delivery Chip*.** To fabricate the on-chip wells, p-1000 pipette tips (Thermo Fisher Scientific Inc.) were positioned over the well channel inlets on the photoresist mold of the “flow layer” and these tips were secured to their positions via cured PDMS. PDMS was then poured within the PMMA barrier to create the bulk PDMS piece that would carry the device’s flow layer and population input wells.
(TIF)

Figure S2 **Description of the earlier generations of the *Population Delivery Chip* and the changes made to eliminate population mixing.** A) Earlier generation device with macro-scale image of the hard-polymer gasket and the PDMS chip. In this device, samples were loaded in wells inside the white polymer gasket, which was sealed to an air pressure line. The bottom of each well linked to the microfluidic chip’s inlets via metal couplers. Leakage at the metal coupler-polymer interface became problematic. B) Final generation device. For parts A and B of this figure, the blue dashed lines surround four equivalently functioning valves in both devices. The yellow arrows in the zoomed-in photos illustrate the difference between the well channel-main channel interfaces in the un-staggered and staggered well channel arrangements. In the earlier version, experiments with colored dyes revealed unintended flow between well channels, hinting at the potential for cross contamination between populations during automated delivery, which was later confirmed. The final generation showed similar potential for mixing in dye experiments, but the placement of valves near the main channel, the addition of the *Main* *Channel* *Flush*, and sequence optimization eliminated population mixing during delivery.
(TIF)

Figure S3 **Unintended worm transport in the previous device iteration.** Two sequential frames are shown from a video of the previous device in action. The blue arrow shows the direction of intended flow in the device as a population is delivered to the main channel. A single worm manages to swim from the bulk population into another well channel (red arrow) instead of the device exit.
(TIF)

Figure S4 **A non-optimized automated delivery sequence causing population mixing.** A) During population delivery from *Well 1* (yellow arrow), *V6* needs to be opened, also allowing *Well* 3’ s population to possibly swim (small red arrow) closer to downstream valve (*V1*), which is closed. B) If we choose to initiate delivery from *Well 7* (yellow arrow) following delivery from *Well 1*, without a flushback step on *Well 3*, we run the risk of *Well* 3’ s worms (small red arrow) also swimming into the main channel with *Well* 7’s population since *V1* must open.
(TIFF)

Figure S5 **Fluid flow rates as a function of applied pressure.** Measured fluid flow rates through *Wells 2, 8, 10*, and *16* plotted against calculated values (dashed lines).
(TIF)

Movie S1 **Slow delivery of worms (3 psi at the gasket and *Main Channel Flush*) with a modified sequence.** To clearly show the worms’ length scale and behavior in the device and highlight the activity of key functional areas of (main channel/well channel intersections, *Exit* *Flush*), worms were delivered in this video at low pressures (3 psi) in a slightly different order than our established sequence for 16 wells.
(MP4)

Movie S2 **A real-time video of the fully automated delivery sequence from 16 wells at the maximum operational pressures.** The order of wells delivered follows the optimized sequence for fast and mixing-free population delivery. Note, many worms move so fast that they are difficult see at the frame rate.
(MP4)

Table S1 **Timings for automated delivery sequence applied to each well and device truth table for delivery from *Well 1*.** “1” indicates the valve/fluid reservoir is pressurized, while “0” means that it is not pressurized. MCF- *Main* *Channel* *Flush*, EF- *Exit* *Flush*. All other valves not described here remain closed throughout this example.
(DOCX)
