# Supplementary figures and images for: An Automated Microfluidic Multiplexer for Fast Delivery of C. elegans Populations from Multiwells
Source: PLoS One. 2013 Sep 17;8(9):e74480. doi: 10.1371/journal.pone.0074480 (PMC3775957; doi:10.1371/journal.pone.0074480)

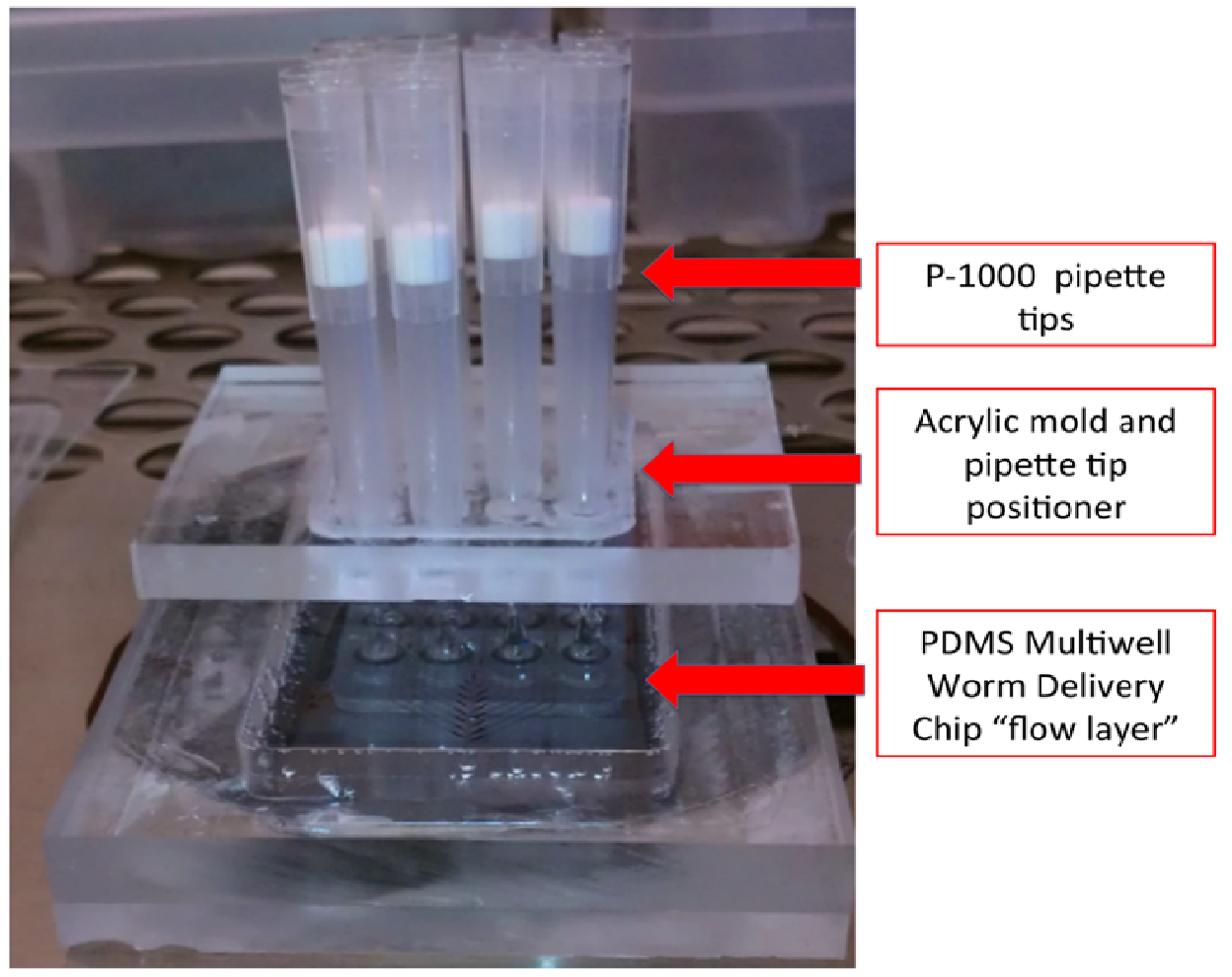

Supplement: Figure S1 — Fabrication of well plate reservoirs for the Population Delivery Chip. To fabricate the on-chip wells, p-1000 pipette tips (Thermo Fisher Scientific Inc.) were positioned over the well channel inlets on the photoresist mold of the “flow layer” and these tips were secured to their positions via cured PDMS. PDMS was then poured within the PMMA barrier to create the bulk PDMS piece that would carry the device’s flow layer and population input wells. (TIF) [file pone.0074480.s001.tif]

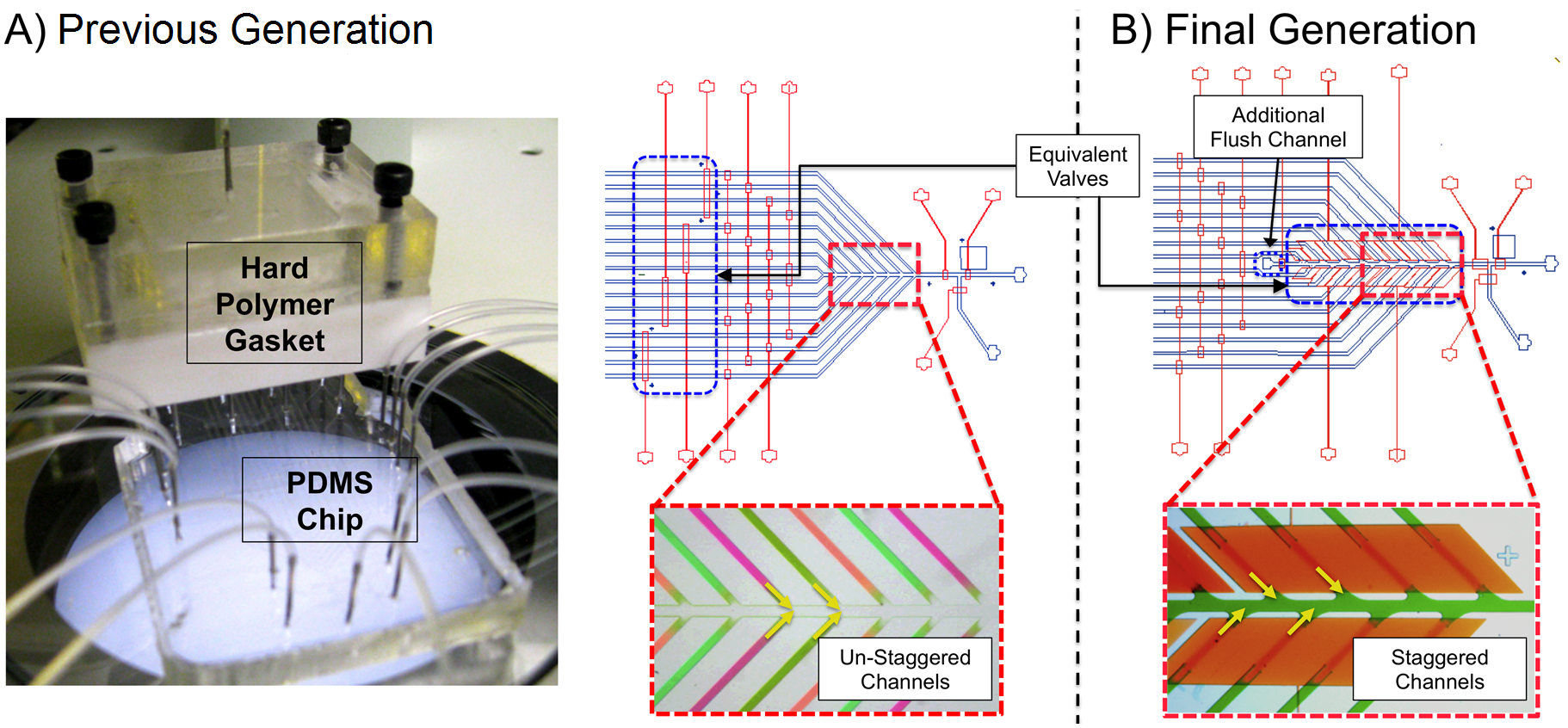

Supplement: Figure S2 — Description of the earlier generations of the Population Delivery Chip and the changes made to eliminate population mixing. A) Earlier generation device with macro-scale image of the hard-polymer gasket and the PDMS chip. In this device, samples were loaded in wells inside the white polymer gasket, which was sealed to an air pressure line. The bottom of each well linked to the microfluidic chip’s inlets via metal couplers. Leakage at the metal coupler-polymer interface became problematic. B) Final generation device. For parts A and B of this figure, the blue dashed lines surround four equivalently functioning valves in both devices. The yellow arrows in the zoomed-in photos illustrate the difference between the well channel-main channel interfaces in the un-staggered and staggered well channel arrangements. In the earlier version, experiments with colored dyes revealed unintended flow between well channels, hinting at the potential for cross contamination between populations during automated delivery, which was later confirmed. The final generation showed similar potential for mixing in dye experiments, but the placement of valves near the main channel, the addition of the Main Channel Flush, and sequence optimization eliminated population mixing during delivery. (TIF) [file pone.0074480.s002.tif]

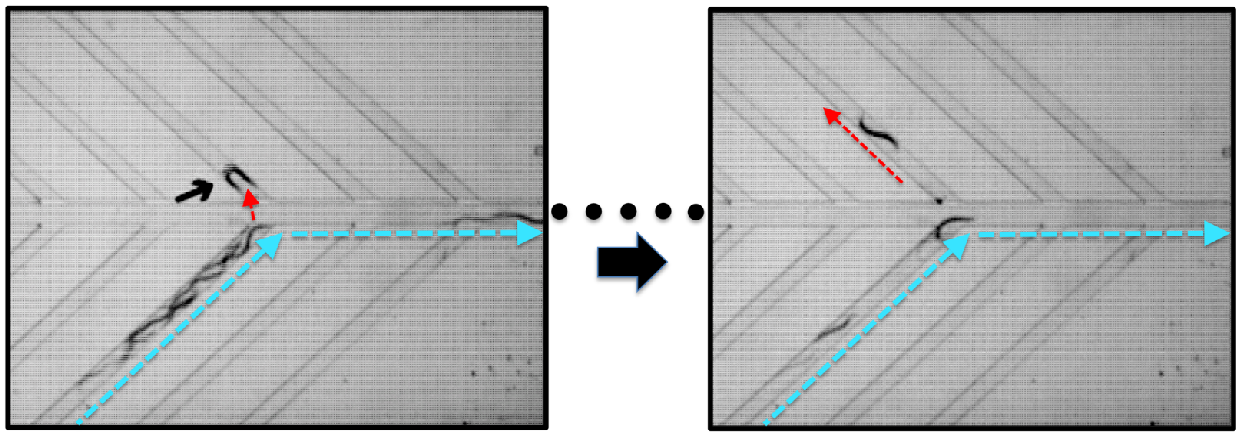

Supplement: Figure S3 — Unintended worm transport in the previous device iteration. Two sequential frames are shown from a video of the previous device in action. The blue arrow shows the direction of intended flow in the device as a population is delivered to the main channel. A single worm manages to swim from the bulk population into another well channel (red arrow) instead of the device exit. (TIF) [file pone.0074480.s003.tif]

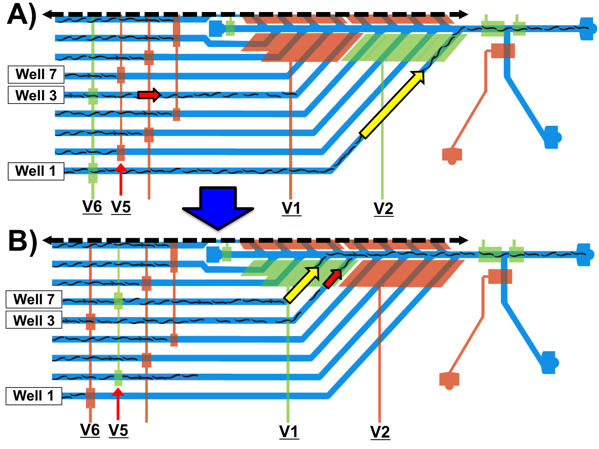

Supplement: Figure S4 — A non-optimized automated delivery sequence causing population mixing. A) During population delivery from Well 1 (yellow arrow), V6 needs to be opened, also allowing Well 3’ s population to possibly swim (small red arrow) closer to downstream valve (V1), which is closed. B) If we choose to initiate delivery from Well 7 (yellow arrow) following delivery from Well 1, without a flushback step on Well 3, we run the risk of Well 3’ s worms (small red arrow) also swimming into the main channel with Well 7’s population since V1 must open. (TIFF) [file pone.0074480.s004.tiff]

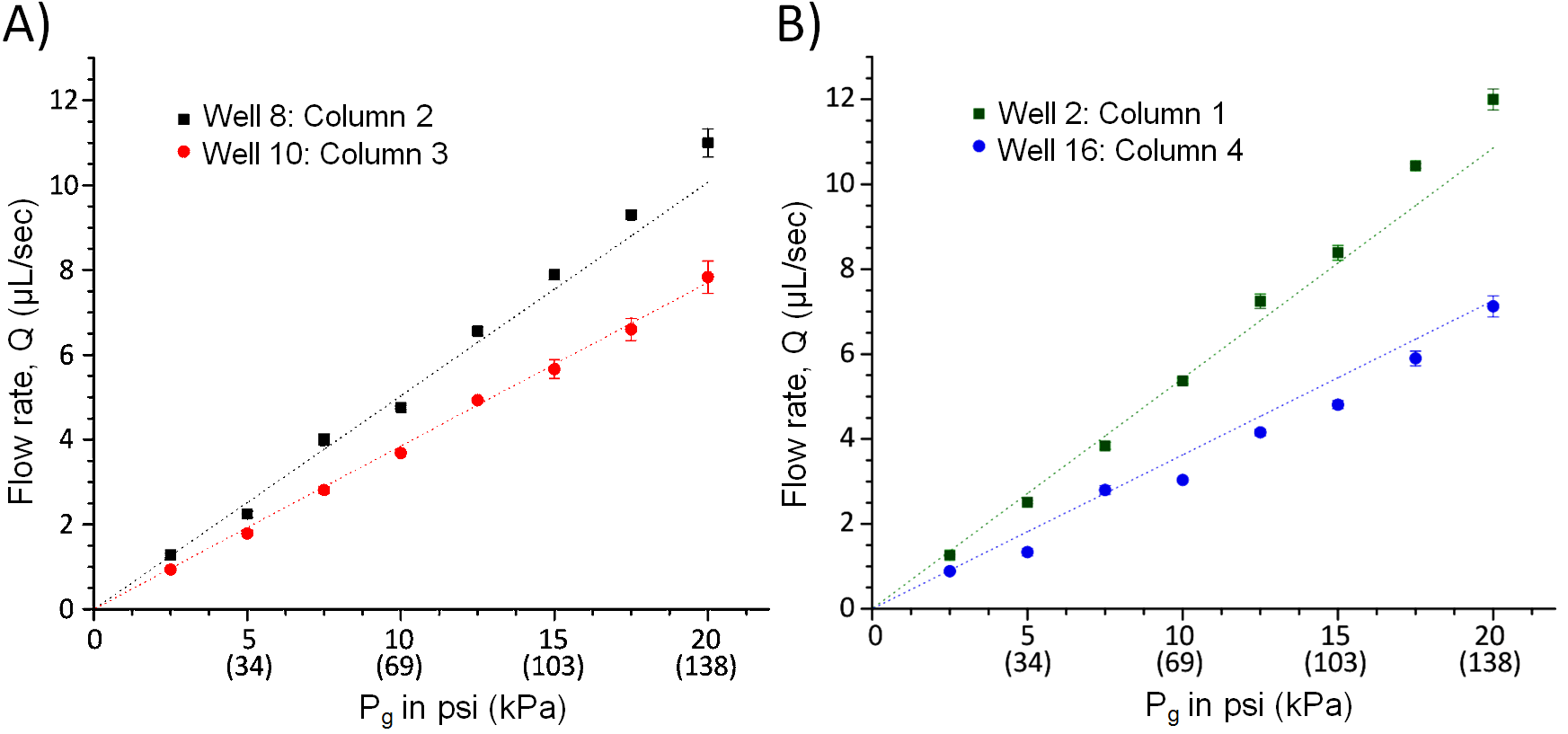

Supplement: Figure S5 — Fluid flow rates as a function of applied pressure. Measured fluid flow rates through Wells 2, 8, 10, and 16 plotted against calculated values (dashed lines). (TIF) [file pone.0074480.s005.tif]
