# Supplementary material for: An Automated Microfluidic Multiplexer for Fast Delivery of C. elegans Populations from Multiwells
Source: PLoS One. 2013 Sep 17;8(9):e74480. doi: 10.1371/journal.pone.0074480 (PMC3775957; doi:10.1371/journal.pone.0074480)
Supplement: Table S1 — Timings for automated delivery sequence applied to each well and device truth table for delivery from Well 1. “1” indicates the valve/fluid reservoir is pressurized, while “0” means that it is not pressurized. MCF- Main Channel Flush, EF- Exit Flush. All other valves not described here remain closed throughout this example. (DOCX) [file pone.0074480.s008.docx]

**Supplemental Table S1: Timings for automated delivery sequence applied to each well and device truth table for delivery from *Well 1*.** “1” indicates the valve/fluid reservoir is pressurized, while “0” means that it is not pressurized. MCF- *Main Channel Flush*, EF- *Exit Flush*. All other valves not described here remain closed throughout this example.

| **Step** | **Timing (s)** | **Gasket** | **V2** | **V6** | **V9** | **V10** | **V11** | **V12** | **MCF** | **EF** |
| --- | --- | --- | --- | --- | --- | --- | --- | --- | --- | --- |
| 1 | 2.8 | 1 | 0 | 0 | 0 | 1 | 0 | 0 | 1 | 0 |
| 2 | 0.5 | 0 | 1 | 1 | 0 | 1 | 0 | 0 | 1 | 0 |
| 3 | 0.7 | 0 | 1 | 1 | 1 | 0 | 0 | 1 | 0 | 1 |
| 4 | 0.7 | 0 | 0 | 0 | 0 | 0 | 1 | 1 | 0 | 1 |
